# Systems-level insights into bronchopulmonary dysplasia from meta-analysis of genome-scale studies

**DOI:** 10.1186/s12931-026-03787-z

**Published:** 2026-07-15

**Authors:** Chris Happs, Prerna Khanna, Charlotte Scoynes, Nelly Muriungi, Akira J. T. Alexander, Josh A. Rogers, Elizabeth Lake, Maximilian Pickup, Nicholas Parkinson, Jonathan E. Millar, J. Kenneth Baillie, Sara Clohisey Hendry

**Affiliations:** 1 Roslin Institute, University of Edinburgh, Edinburgh, UK; 2 Institute for Regeneration and Repair, Baillie-Gifford Pandemic Science Hub, University of Edinburgh, Edinburgh, UK; 3 Royal (Dick) School of Veterinary Studies, University of Edinburgh, Edinburgh, UK

**Keywords:** Bronchopulmonary Dysplasia, Acute Respiratory Distress Syndrome, Adaptive Immunity, T-cells, Lymphoid cells, Leukocyte cells

## Abstract

**Background:**

Despite marked improvements in survival following preterm birth, the incidence of bronchopulmonary dysplasia (BPD) remains the most prevalent complication of prematurity and carries the risk of long-term morbidity. Characterising the cellular and molecular mechanisms driving disease progression is critical for informing clinical management and improving outcomes. To this end, we conducted a meta-analysis of genome-scale studies to identify molecular pathways implicated in BPD progression in both human cohorts and animal models.

**Methods:**

Gene lists associated with BPD in humans, and in rodent models, were extracted from systematically identified genome-scale studies. These gene lists were subsequently analysed using the meta-analysis by information content (MAIC) algorithm, which integrates multiple datasets to generate a single aggregated, ranked gene list based on the cumulative strength of evidence for each gene. Comparative analyses were then performed between the human and rodent BPD datasets, as well as between the human BPD dataset and our previously generated Acute Respiratory Distress Syndrome MAIC dataset.

**Results:**

Across all analyses, a consistent enrichment of genes involved in leukocyte-mediated antigen presentation and lymphocyte development and activation was observed, suggesting a shift from acute innate immune injury toward a more sustained lymphoid-driven inflammatory process during BPD progression. Comparative analyses revealed limited overlap between BPD and ARDS gene sets, suggesting divergent disease mechanisms, while still highlighting shared immune activation pathways. Human–rodent comparisons showed divergence in tissue remodelling signatures, likely reflecting differences in sample sources, yet converged on key conserved signals such as CD3E and IL1R2, implicating common inflammatory regulatory mechanisms.

**Conclusions:**

MAIC effectively identifies conserved molecular signatures in BPD highlighting lymphoid lineage signatures that are not readily apparent in the primary data. These signatures offer insights relevant to immune-modulatory therapeutic strategies, highlighting key processes involved in antigen presentation and modulation of T-cell activation and development during the progression of Bronchopulmonary Dysplasia.

**Supplementary Information:**

The online version contains supplementary material available at https://doi.org/10.1186/s12931-026-03787-z.

## Background

Bronchopulmonary dysplasia (BPD), a chronic lung disease, is one of the most common complications of preterm birth, affecting up to 30% of preterm infants born before 32 weeks gestational age (GA) [1–3]. Antenatal (e.g. choriamnionitis, intrauterine growth restriction, preeclampsia) and postnatal (e.g. mechanical ventilation, supplemental oxygen, infection) factors disrupt the developing lung leading to decreased alveolarisation, larger alveoli, irregular pulmonary vessels, and fibrotic tissue [3, 4]. The effects of BPD can be life-long; individuals with BPD are more likely to require rehospitalisation during their childhood and to have limited lung function compared to those born preterm without BPD [5–8].

Understanding the molecular mechanisms that contribute to BPD could aid in risk prediction, developing targeted therapies, and understanding disease mechanisms. The heterogeneity of the disease has hampered efforts to identify reliable, consistent biomarkers and causative genes associated with disease progression. Alongside evidence from twin studies pointing to a possible heritable component in BPD some putative genetic candidates have been identified (e.g. SPOCK2, CRP) [9–11] though no gene has been significantly associated with the development of BPD by genome-scale studies such as Genome Wide Association Studies (GWAS) or exome sequencing. Recently, transcriptomic data has been used to identify four endotypes of BPD, distinguished by T-helper cell and T-cell signalling [12].

Comparative analyses across species can provide valuable insights into common mechanisms while also highlighting potential species-specific responses across models. Rodent models have been widely employed to study the pathophysiology of BPD and have concentrated primarily on hyperoxia-induced lung injury, mimicking the impaired alveolar development and inflammation observed in preterm infants [13]. Anatomical and physiological differences have prevented in-depth research of ventilator induced lung injury in this model.

By integrating heterogeneous data sources, we can gain new perspectives. We conducted two concurrent systematic reviews of the BPD literature, focusing on genome-scale studies in humans and animal models. Results were extracted from identified studies, where possible, and analysed using the Meta Analysis by Information Content (MAIC) algorithm. This is a previously described algorithm that uses data-driven gene list weightings to produce a comprehensive ranked list of genes associated with the trait of interest. The primary purpose of the MAIC algorithm is to identify core biological drivers by integrating diverse experimental modalities. The algorithm treats each method as a primary source of evidence allowing for the prioritisation of genes based on the cumulative strength of evidence across multiple levels of biological regulation. To identify core biological drivers of lung injury, we compared our results with a well-characterised ARDS MAIC dataset. This allowed us to identify genes representing common pathways of lung injury and remodelling, effectively filtering the noise of transient developmental signals.

Our findings show that genes associated with development and maintenance of adaptive immune cells feature prominently, suggesting that adaptive-lineage cells may play a larger role in BPD pathogenesis, and potentially a wider role in ventilator associated lung injury, than previously appreciated.

## Methods

### Systematic review

The systematic review and meta-analysis protocol was registered with the International Prospective Register of Systematic Reviews (PROSPERO; CRD42022306270, CRD42024550229). The review is reported in compliance with the Preferred Reporting Items for Systematic Reviews and Meta-Analyses (PRISMA) guidelines [14].

### Search strategy and selection criteria

A detailed description of our search strategy and eligibility criteria is provided in the Supplementary Methods. Briefly, we searched MEDLINE and Embase without language restrictions on 24/05/2024. We included human genome-scale studies reporting associations between genes, transcripts, or proteins. For human studies we accepted any contemporaneous BPD definition. For rodent studies, author defined BPD study groups were accepted. We excluded candidate in vivo or in vitro studies (< 50 genes/transcripts/proteins), candidate gene associations, and studies with < 5 patients per arm. Following deduplication, titles were initially screened using Screenatron [15].

Abstracts were then screened against eligibility criteria, this included organising the studies into separate collections of human and animal studies, with an independent author resolving inconsistencies. Gene list is used as shorthand, though lists could also be made up of transcripts and proteins. Full texts were retrieved and analysed for inclusion before extraction of gene lists for inclusion in MAIC. Input lists were processed as previously described [16–18]. Briefly, lists were considered ranked if metrics of statistical significance (p-value) and/or fold change (FC) were reported. These lists were ordered by p-value/false discovery rate (low to high) then, where applicable, by absolute fold change or effect size (high to low). Gene names were converted to HGNC gene symbols (or Ensembl/Refseq symbols if no HGNC symbol). Although two non-rodent studies met the inclusion criteria, the predominance of rodent-based studies led us to focus exclusively on rodents in subsequent analyses. Rodent genes were mapped to human gene symbols (HGNC where available) using the NCBI Homologene database (downloaded 10/06/2020) via custom Python scripts. Where no human homologue was identified genes were omitted from the lists as our intention was a comparative study. For rodent genes with more than one candidate human homologue, the candidate homologue list was first narrowed by excluding human gene symbols already mapped to a higher entry in the rodent gene list, and if this failed to resolve the ambiguity then the gene was mapped to the first candidate human gene symbol alphabetically. Where multiple rodent genes mapped to the same human gene, only the highest-ranked rodent gene was retained. We acknowledge that this system has limitations. However, ensuring unique list entries across species is a difficult constraint, and there is currently no method to fully address this.

#### MAIC

The MAIC algorithm has previously been validated and described in detail [16–20]. A full description and the source code is available at https://baillielab.net/maic. We implemented pymaic v0.2 in Python v3.9 and used Technique to categorise input lists. MAIC combines both ranked and unranked lists, of unknown quality, to build a comprehensive ranked list of entities according to 4 basic assumptions.1. There is a set of true positives (genes implicated in BPD), 2. A gene is more likely to be a true positive if it appears in datasets from more than one source, 3. A gene is more likely to be a true positive if it appears in datasets with a higher proportion of replicated genes. 4. A gene is more likely to be a true positive if it appears in datasets from multiple methods or modalities. This approach is made possible by using the gene symbol as the primary unit of analysis, allowing the algorithm to collate evidence regardless of whether the original experiment measured DNA variation, RNA expression or protein abundance.

The results are ranked by MAIC score. The score is calculated by the MAIC algorithm as follows: The initial MAIC score for each gene is set to 1. A gene receives a higher score if it is found in many different types of data categories (e.g., different Techniques) rather than just appearing frequently within a single category. To ensure that no single type of data category dominates the results for a single gene, the system takes only the single strongest piece of evidence for a gene from each available data category. The final score is the sum of these top values from every category. If a gene is missing from a specific category, it contributes zero to the total. Consequently, a gene with evidence from both a genomic and proteomic study will rank higher than a gene appearing in multiple studies of the same modality, as this demonstrates cross-modal biological robustness. A more comprehensive description can be found at https://baillielab.net/maic/method. The unit invariant knee method was used to identify the inflection point in the MAIC score curve and thus prioritise genes for further investigation.

### Functional analyses and tissue expression

#### Protein–protein Interaction network

Prioritised genes were analysed using the online tool STRING (https://string-db.org) to determine potential protein–protein interactions (PPI). Gene symbols were mapped to STRING identifiers from the STRING database using the online portal. Only genes with successfully resolved mappings in the STRING database were brought forward for network construction. For humans 919 gene names were mapped to proteins, for rodents 1726. A PPI network was constructed using the MCL (Markov Clustering) algorithm, with an inflation parameter of 3 and focused on high confidence (interaction score of > = 0.7) interactions. While network graphs represent evidence from all active interaction sources, only coexpression interactions are displayed. The network was exported. Hub proteins were identified using the overlap of the top 100 ranked proteins, ranked using 5 common algorithms (MCC, MNC, Degree, EPC and DMNC) using CytoHubba 0.1 implemented in CytoScape 3.10.3.

#### Results systematic review

We first conducted a systematic review of studies that reported associations between genes, transcripts, or proteins, and the development of BPD in humans or the study of BPD in non- human mammalian models (Supplementary Methods). We did not restrict the search terms to a specific definition of BPD; however, the timeline of genome-scale studies results in an indirect emphasis on more recent BPD definitions. [2] Our search yielded 4450 unique citations that were evaluated for inclusion (Supplementary Figs. 1 and 2). For full text evaluation we retrieved 76 articles for human studies and 118 for mammalian studies, without overlap. Although large, non-human mammalian models are used to understand BPD progression, [21, 22] we did not identify any that met our inclusion criteria (Supplementary Tables 1 and 2) and further analysis therefore focused on rodent models, such as mice and rats.

#### Meta-analysis by information content of human genome-scale bronchopulmonary dysplasia studies

**Fig. 1:**
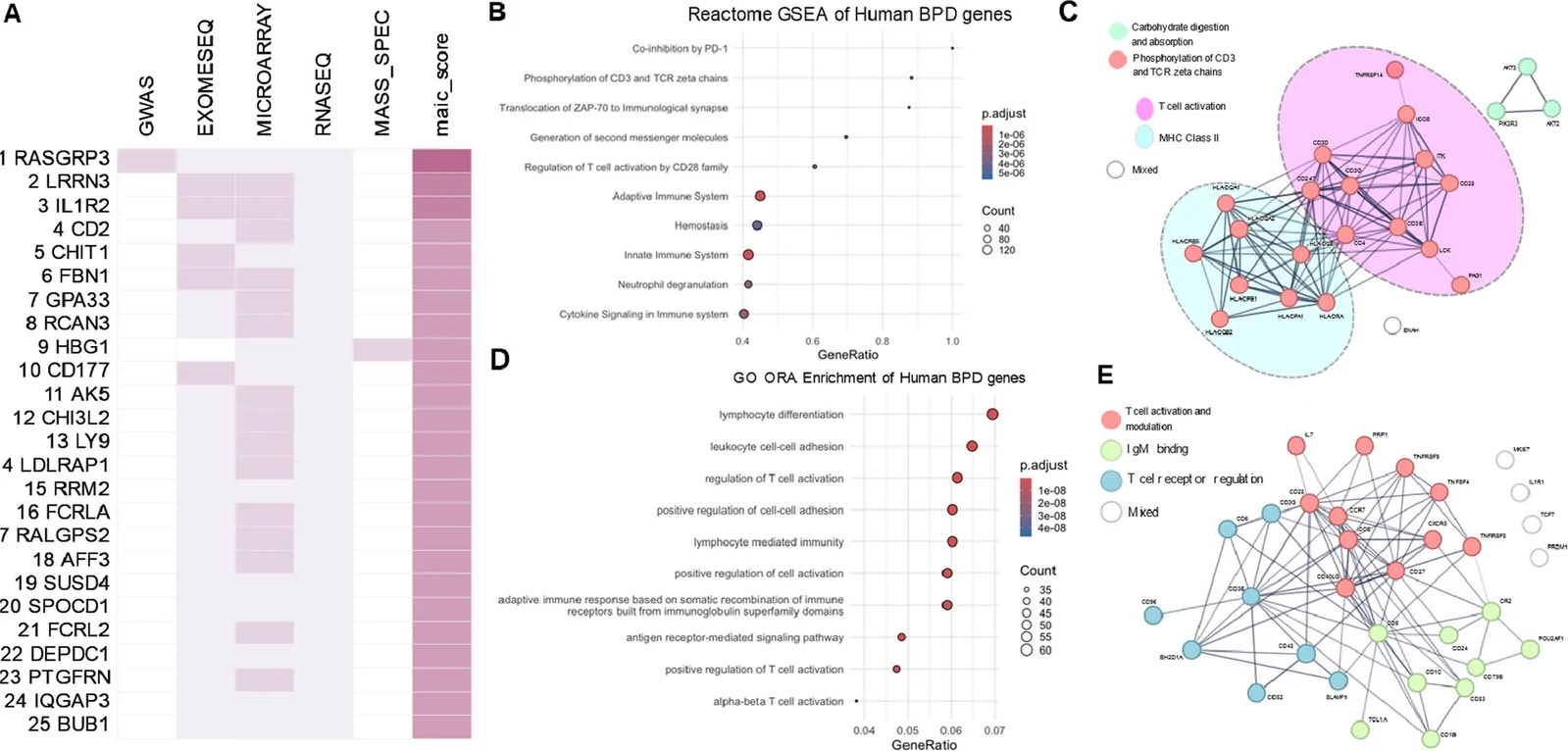
Meta-Analysis by information Content of human genome-scale Bronchopulmonary Dysplasia (BPD) studies. **A** Heatmap of top 25 genes ranked by MAIC score and indicating the source of the data. **B** Gene set enrichment analysis (GSEA) of the human BPD gene set against the Reactome database. Size of circle represents the number of genes in the pathway. The colour is reflective of adjusted p-value (p.adjust) as indicated in the legend. The Gene Ratio reflects the proportion of input genes involved in each pathway. **C** A core set of 23 proteins clustered using MCL (Markov clustering) resulting in 2 clusters: carbohydrate digestion and absorption (green) and phosphorylation of CD3 and T-cell receptor zeta chains. The second manually annotated cluster has features of MHC signalling (blue) and T-cell activation (purple). **D** Over-representation analysis (ORA) of Gene Ontology (GO) terms across all ontologies for prioritised human BPD genes. The colour is reflective of adjusted p-value (p.adjust) as indicated in the legend. The Gene Ratio reflects the proportion of input genes involved in each pathway. **E** A protein–protein interaction network of 32 hub proteins clustered using MCL clustering. Clusters are coloured and named according to the legend, pathway names have been manually annotated for clarity

From 76 human studies, 21 met our eligibility criteria (Supplementary Fig. 1, Table 1), yielding 31 gene lists (23 ranked and 8 unranked) employing 5 experimental techniques (Supplementary Table 3). The included studies represented 4358 infants (cases = 2247, controls = 2111). While the definition of BPD varied between studies, over two-thirds of studies, (71.4%, 15/21), used receipt of respiratory support at 36 weeks GA (Table 1). All but a single study used primary tissue or blood [38]. MAIC collated and ranked 8017 unique genes across the datasets (Supplementary Table 4), and the top 25 are shown here (Fig. 1A; Top 100 in Supplementary Fig. 3 A). A single gene was supported by evidence from 4 experimental techniques (*RASGRP3*, rank = 1). Few genes (*n* = 53 genes, 0.6%) were supported by > = 3 methods while 745 genes (9.74%) were supported by more than 1 method. In the top 10 ranked genes we observe immune regulation (*RASGRP3, CD2, GPA33*, *IL1R2, CD177)* and extracellular structure *(CHIT1, RCAN3, LRRN3, FBN1)*. Additionally, a subunit of foetal haemoglobin, *HGB1,* features. Although the top hit (*RASGRP3*) is supported by data from GWAS experiments, the methodology overall contributed very little information content to the top ranked genes (Supplementary Fig. 3B). Cell type–specific enrichment analysis indicates that the gene rankings are most strongly driven by cells of the lymphoid lineage (Supplementary Fig. 4).

**Table 1:** Characteristics of studies included in Human BPD MAIC analysis

Study	Year	Type	Technique	Tissue	Number of participants	Time sample analysed	BPD Definition
Ahmed et al*.,* [23]	2024	Proteomic	MassSpec	Oral	17	3rd week of life	Resp. Supp at 36 weeks GA
Ambalavanan et al*.,* [24]	2015	Genomic	GWAS	Blood	834	Earliest blood spot test	Resp. Supp at 36 weeks GA
Bhattacharya et al*.,* [25]	2012	Transcriptomic	Microarray	Lung	11	At autopsy	Resp. Supp at 36 weeks GA
Bhattacharya et al*.,* [26]	2021	Transcriptomic	RNAseq	Blood	145	At hospital discharge	Resp Supp at 3,6,9,12 months
Da paepe et al*.,* [27]	2010	Transcriptomic	Microarray	Lung	6	At autopsy	Ventilator dependent for entire lifespan
Dai et al*.,* [28]	2021	Genomic	ExomeSeq	Blood	245	Within 7 days after birth	Rest Supp on 1 st day of life, < 32 weeks GA
Fulton et al*.,* [29]	2018	Transcriptomic	Microarray	Tracheal aspirates	13	Within 7 days after birth	Resp. Supp at 36 weeks GA
Gong et al*.,* [30]	2020	Transcriptomic	Microarray	Blood	40	Day 28	Resp. Supp at 36 weeks GA
Hadchouel et al*.,* [10]	2011	Genomic	GWAS	UCB	418	Postnatal (not specified)	Resp. Supp at 36 weeks GA
Hamvas et al*.,* [31]	2018	Genomic	ExomeSeq	Saliva	765	Postnatal (not specified)	Resp. Supp at 36 weeks GA
Li et al*.,* [32]	2015	Genomic	ExomeSeq	Blood	50	Blood spot test	Resp. Supp at 36 weeks GA
Luo et al*.,* [33]	2023	Genomic	ExomeSeq	Oral	66	Not specified	Resp. Supp at 36 weeks GA
Oji Mmuo et al*.,* [34]	2021	Transcriptomic	RNAseq	Tracheal aspirates	55	Following routine suctioning	Resp. Supp at 36 weeks GA
Pietrzyk et al*.,* [35]	2013	Transcriptomic	Microarray	Blood	111	Postnatal Day 5, 14, 28	Resp. Supp and birth < 32 weeks GA
Siddaiah et al*.,* [36]	2023	Multiomic	RNAseq	Tracheal aspirates	46	Postnatal (not specified)	Resp. Supp at 36 weeks GA
Torgerson et al*.,* [37]	2018	Genomic	GWAS	N/A	511	Postnatal Day 7 to 21	Resp. Supp at 36 weeks GA
Tsotakos et al*.,* [38]	2022	Transcriptomic	RNAseq	Cultured cells—A549	N/A	In vitro	NA—cultured cells
Wang et al*.,* [39]	2013	Genomic	GWAS	Blood	1726	Blood spot test	Resp. Supp at 36 weeks GA
Windhorst et al*.,* [40]	2023	Transcriptomic/Proteomic	Microarray	UCB	30	At birth	O_2_ > 21% for 28 days by 36 weeks
Yuon Chou et al*.,*	2023	Transcriptomic	RNAseq	UCB	109	Postnatal Day 14 and 28	Resp. Supp at 36 weeks GA
Zasada et al*.,* [41]	2019	Proteomic	MassSpec	UCB	57	At birth	Resp. Supp at 36 weeks GA

*UCB* Umbilical Cord Blood, *GA* Gestational age, *Resp. Supp* Respiratory support, *Oral *Oral mucosa

#### Enrichment analyses of Human BPD MAIC gene set

Gene set enrichment analysis (GSEA) using the Reactome database identified 75 significantly enriched pathways (p.adjust < 0.05), highlighting multiple aspects of T-cell activation (Fig. 1B). This result, alongside signals from various aspects of immunity, was substantiated by searches of other databases (Supplementary Materials). The signal was primarily driven by a core set of 23 genes (Supplementary Fig. 3 C), which the Markov Cluster Algorithm (MCL) partitioned into two distinct modules (Fig. 1C): one linked to carbohydrate digestion and absorption and a second associated with CD3 and TCR zeta-chain phosphorylation. The latter constituted the substantially larger module, encompassing multiple T-cell receptor components as well as several elements of the HLA complex.

We prioritised 945 genes for further investigation (Supplementary Fig. 3D), as previously described and of these 310 (38%) were identified as druggable (Supplementary Materials), according to The Druggable Genome [42]. We performed Over-representation Analysis (ORA), on the prioritised gene set. This identified a total of 154 significant Gene Ontology terms (p.adjust < 0.05), while the Reactome database replicated much of the signal observed in the GSEA (Supplementary Materials). We grouped ORA results from the Gene Ontology database (encompassing all ontologies) first based on semantic similarity, and then based on function (Fig. 1C) [43] which reveals an over-representation of immune system processes associated with lymphoid-mediated immunity, particularly regulation of T-cell activation.

#### Protein–protein Interaction network of Human BPD MAIC gene set

We created a protein–protein interaction (PPI) network using the prioritised gene set. Clustering of this network using MCL identified 25 clusters with > = 5 members. The 7 largest clusters contained > = 10 members (Supplementary Fig. 5 A-E shows the top 5 clusters). Separately, using previously reported methods (Millar et al., 2024), we identified 32 hub proteins likely to play central roles in the broader PPI network (Supplementary Fig. 5 F). MCL clustering of this hub revealed three major clusters related to T-cell function, corresponding to activation (red, blue), receptor regulation (blue), and immunoglobulins (green) (Fig. 1E). Overall, our MAIC analysis identified a robust lymphoid cell signature across a diverse range of human genome-scale studies of BPD.

#### Comparative analysis with ARDS MAIC gene set

Acute lung injury caused or worsened by mechanical ventilation during treatment is a feature of BPD in neonates and Acute Respiratory Distress Syndrome (ARDS) in adults and children. We wished to identify both shared and distinct factors underlying each syndrome. We evaluated the human BPD MAIC dataset against our previously generated ARDS MAIC analysis [17].

**Fig. 2:**
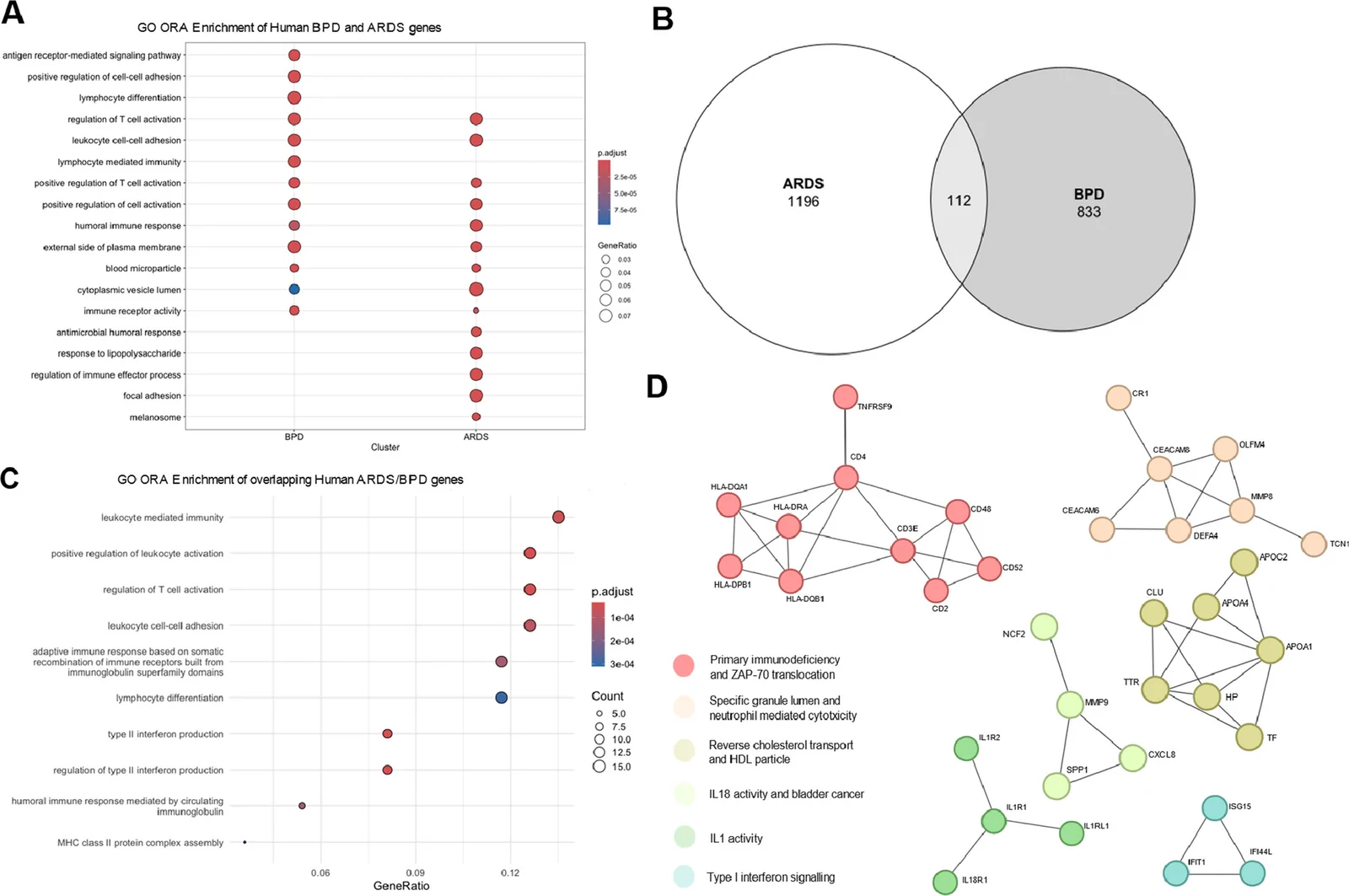
Comparison of analyses between Acute Respiratory Distress Syndrome (ARDS) and Bronchopulmonary Dysplasia (BPD). **A** The top 10 significantly over-represented terms from over-representation analysis (ORA) of Gene Ontology (GO) terms across all ontologies for prioritised BPD (left) and ARDS (right) genes demonstrating overlap. The colour is reflective of adjusted p-value (p.adjust) as indicated in the legend. The size of the circle reflects the Gene Ratio. **B** Euler plot showing overlap of prioritised genes between BPD and ARDS MAIC analyses. **C** Over-representation analysis of GO terms across all ontologies for ARDS/BPD overlap genes. Size of circle represents the number of genes in the pathway. The colour is reflective of adjusted p-value (p.adjust) as indicated in the legend. The Gene Ratio reflects the proportion of input genes involved in each pathway. **D** A protein–protein interaction network prioritised proteins that are shared between ARDS and BPD gene sets, clustered using MCL (Markov) clustering. Clusters are coloured and named according to the legend

Focusing on the prioritised genes from both datasets, ORA highlighted shared pathology relating to regulation of T-cell activation alongside leukocyte cell activation (Fig. 2A). There were 112 genes that overlapped in the prioritised gene set lists, (Fig. 2B). This overlap was found to be significant (p < 0.005) and occurred primarily toward the top of the ranked lists (Supplementary Fig. 6 A). Over-representation analysis further highlighted leukocyte-mediated immunity, lymphocyte-mediated immunity, and positive regulation of both T-cells and type II interferon in the overlapping genes (Fig. 2C). A PPI network focused on the overlap between datasets identifies 6 clusters of > = 3 proteins (Fig. 2D), the largest of which (red) reflects the lymphocyte signal seen elsewhere in our analysis.

#### Meta-Analysis by Information Content of genome-scale rodent bronchopulmonary dysplasia studies

**Fig. 3:**
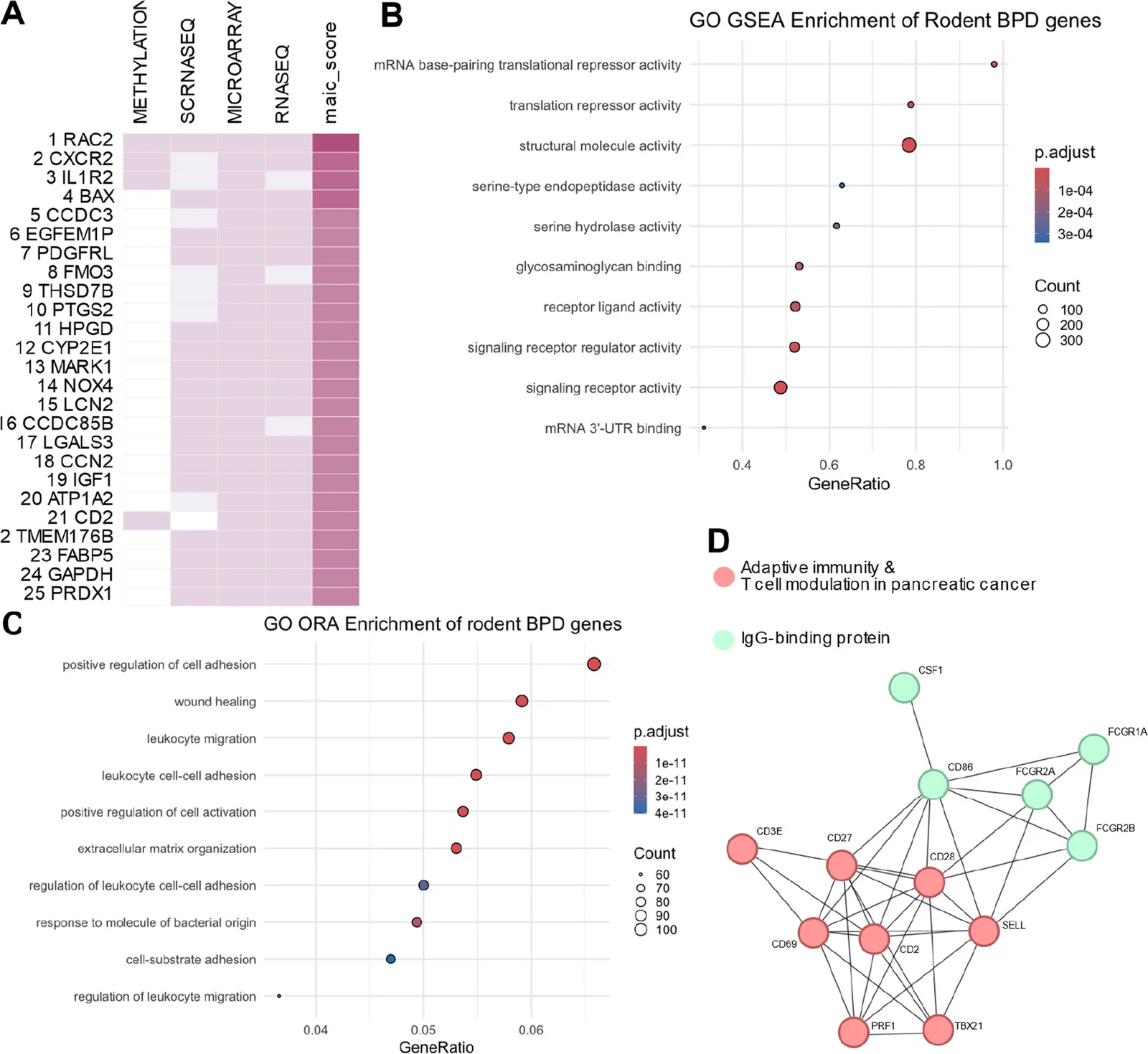
Meta-Analysis by Information Content of rodent genome-scale Bronchopulmonary Dysplasia (BPD) studies. **A** Heatmap of top 25 genes ranked by MAIC score and indicating the source of the data. **B** Gene set enrichment analysis (GSEA) of the rodent BPD gene set. Size of circle represents the number of genes in the pathway. The colour is reflective of adjusted p-value (p.adjust) as indicated in the legend. The Gene Ratio reflects the proportion of input genes involved in each pathway. **C** Over-representation analysis (ORA) Gene Ontology terms across all ontologies for prioritised rodent BPD genes. The colour is reflective of adjusted p-value as indicated in the legend. The Gene Ratio reflects the proportion of input genes involved in each pathway. **D** A protein–protein interaction network (PPI) of 13 hub proteins clustered using MCL clustering. Relevant pathways are shown in the legend

From 20 eligible rodent BPD studies (Supplementary Figure 2, Table 2) we extracted and analysed 35 gene lists (28 ranked and 7 unranked), employing 5 experimental techniques (Supplementary Table 3). Of these studies, 13 used mice models and 7 used rat models. To enable cross-species comparisons, we mapped the genes from both species to their human ortholog symbols as part of the MAIC workflow. The majority (17/20; 85%) of studies modelled BPD by exposing neonatal pups to a hyperoxic environment, though the approaches differed. For example, there was variation in the timeframe of hyperoxia (from 3 to 14 days), recovery in room air (0 days to 57 days), continuous versus intermittent hyperoxia, and the percentage oxygen (70–100%) used across the studies. Other methods to model BPD included lipopolysaccharide (LPS) treatment (1 study) and induced IUGR through maternal diet (1 study). MAIC collated 7637 genes across all datasets (Fig. 3A; Supplementary Table 5; Top 100 in Supplementary Fig. 7 A) and the top 3 genes were supported by evidence from > = 4 experimental techniques (*RAC2*, *CXCR2*, *IL1R2*). One in five genes were supported by more than 1 method (n = 1597, 20.91%), with 141 genes (1.85%) supported by > = 3 methods. Immune processes are broadly highlighted within the top ten genes (*RAC2*, *CXCR2*, *IL1R2*, *CCDC3*, *PTGS2*). Notably, IL1R2 appears among the top 10 results in both human (rank = 3) and rodent (rank = 3) BPD MAIC analyses. Gene rankings are strongly driven by fibroblast cells, with far less emphasis on the lymphoid cells seen in the human dataset, according to cell type–specific enrichment analysis (Supplementary Fig. 8).

**Table 2:** Characteristics of studies included in Rodent BPD MAIC analysis. Techniques are outlined in supplementary table X

**Study**	**Technique**	**Animal**	**Tissue**	**Method**
Al Mudares et al*.,* [44]	RNAseq	Mouse	Whole lung	Hyperoxia
Bao et al*.,* [45]	Microarray	Mouse	Whole lung	Hyperoxia
Bhaskaran et al*.,* [46]	Microarray	Rat	Whole lung	Hyperoxia
Bhattacharya et al*.,* [26]	RNAseq	Mouse	Whole lung	Hyperoxia
Chao et al*.,* [47]	Microarray	Mouse*	Whole lung	Hyperoxia
Chen et al*.,* [48]	DNA Methylation	Rat	Whole lung	Hyperoxia
Cheng et al*.,* [49]	RNAseq	Rat	Right lung lobe	Hyperoxia
Coarfa et al*.,* [50]	RNAseq	Mouse	Whole lung	Hyperoxia
Dénervaud et al*.,* [51]	RNA microarray	Rat	Whole lung	Hyperoxia
Na Dong et al*.,* [52]	RNAseq	Rat	Whole lung	Hyperoxia
Dravet Gounot et al*.,* [53]	RNAseq	Rat	Whole lung	Induced IUGR
El Saie et al*.,* [54]	Mass Spec	Mouse	Whole lung	Hyperoxia
Hurskainen et al*.,* [13]	scRNAseq	Mouse	Whole lung	Hyperoxia
Natarajan et al*.,* [55]	Microarray	Mouse*	Whole lung	Hyperoxia
Nichols et al*.,* [56]	QTL analysis	Mouse	Whole lung	NA
Nitkin et al*.,* [57]	RNAseq	Mouse	Whole lung	LPS treated
Rao et al*.,* [58]	RNAseq	Mouse*	Whole lung	Hyperoxia
Revhaug et al*.,* [59]	Microarray	Mouse	Whole lung	Hyperoxia
Scaffa et al*.,* [60]	scRNAseq	Mouse	Whole lung	Hyperoxia
Shrestha et al*.,* [61]	Microarray	Rat	Whole lung	Hyperoxia

Mouse* Mouse line with specific mutation, *NA* No treatment, *IUGR *Intrauterine growth restriction, *LPS* Lipopolysaccharide

#### Enrichment analyses of Rodent BPD MAIC gene set

Gene set enrichment analysis of the rodent MAIC gene set using the Reactome database identified 113 significantly enriched pathways (p.adjust < 0.05), indicating strong signals for mRNA and translation associated pathways (Fig. 3B). Analysis of the ranked genes contributing to this signal shows a cluster of lower ranked genes (rank > 2500).

that drive these signals (Supplementary Fig. 7 C). To fully exploit the power of MAIC, we prioritised the top 1783 genes from the rodent BPD gene set for further investigation (Supplementary Fig. 7D). Over-representation analysis revealed a significant overrepresentation of 992 Gene Ontology terms (p.adjust < 0.05) related to leukocyte cell migration and activation, and extracellular matrix organisation (Fig. 3C). An MCL clustered PPI network identified 55 clusters with > = 5 members (the top 5 are shown in Supplementary Fig. 9). The 21 largest clusters contained > = 10 members. Among these, we found programs associated with the adaptive immune response and mitosis. Using the PPI network we identified 13 hub proteins, including FC gamma receptors (FCGR1A, FCGR2A, FCGR2B) and lymphocyte markers (CD3E, CD2), suggested as being central to the wider network in rodent models of BPD (Fig. 3D).

#### Comparative analysis of Human and Rodent BPD MAIC gene sets

**Fig. 4:**
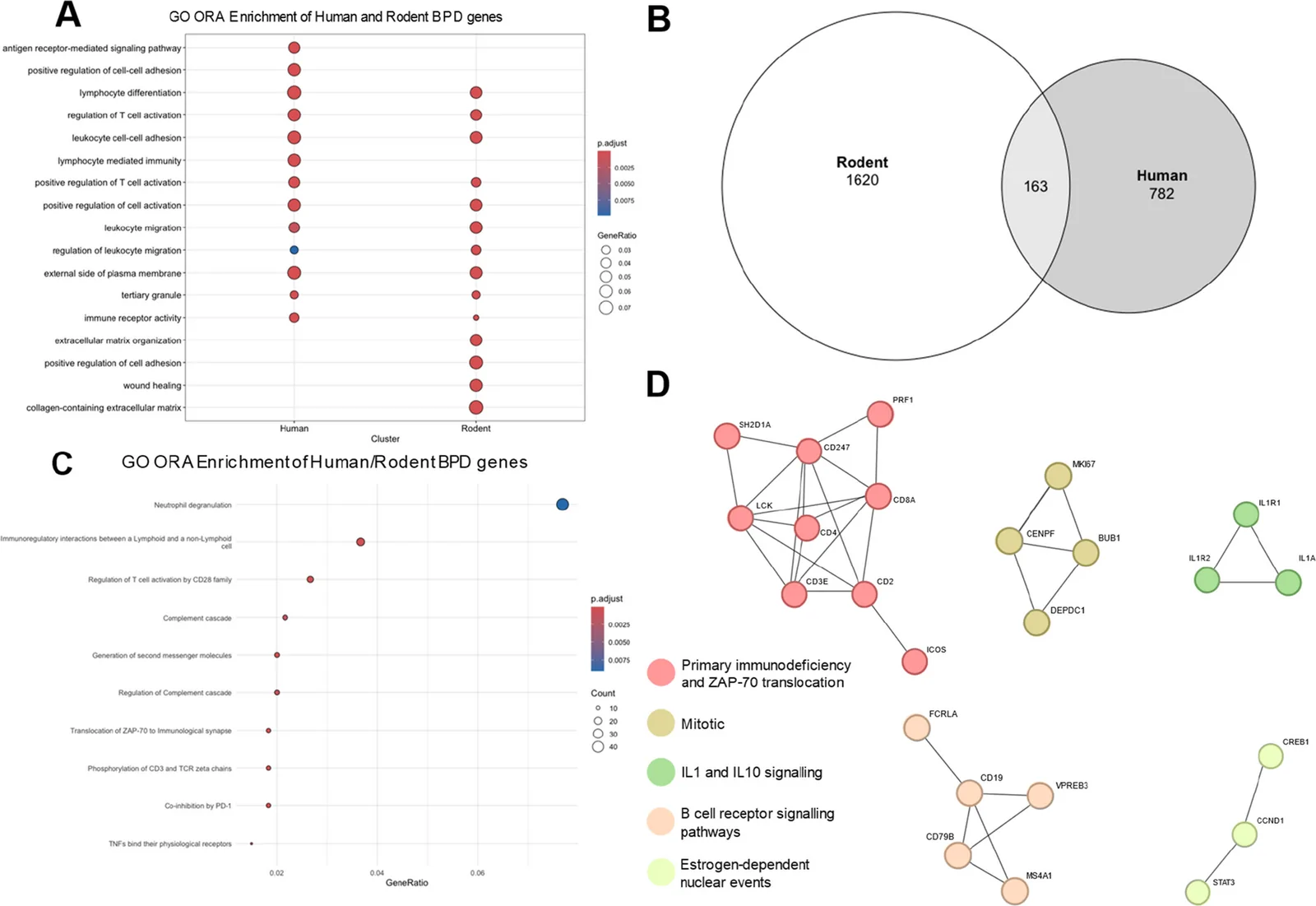
Comparison of MAIC analyses between human and rodent Bronchopulmonary Dysplasia (BPD) datasets. **A** The top 10 significantly over-represented terms from over-representation analysis (ORA) of Gene Ontology (GO) terms across all ontologies for prioritised Human (left) and Rodent (right) BPD genes demonstrating overlap. The colour is reflective of adjusted p-value (p.adjust) as indicated in the legend. The size of the circle reflects the Gene Ratio. **B** Euler plot showing overlap of prioritised genes between Human and Rodent BPD MAIC analyses. **C** Over-representation analysis of GO terms across all ontologies for Human/Rodent overlap BPD genes. Size of circle represents the number of genes in the pathway. The colour is reflective of adjusted p-value (p.adjust) as indicated in the legend. The Gene Ratio reflects the proportion of input genes involved in each pathway. **D** A protein–protein interaction network (PPI) of 24 proteins that are shared between Human and Rodent BPD gene sets, clustered using MCL clustering. This results in 4 clusters, coloured and named according to the legend

Over-representation analysis showed that while the lymphoid signal observed in humans above was seen in the rodent gene set, we also saw the role of the extracellular matrix and wound healing highlighted (Fig. 4A). The gross overlap between the two parallel MAIC analyses carried out in this study was not shown to be significant by hypergeometric test (p > 0.5) (Supplementary Table 6). However, focusing on the prioritised genes from both gene sets indicated the overlap of 163 genes (Fig. 4B) was significant (Supplementary Table 6). and occurred mostly toward the top of the prioritised lists (Supplementary Fig. 6B). Over-representation analysis of shared human and rodent BPD genes showed further enrichment of lymphocyte pathways from prior analyses and additionally supported the neutrophil degranulation and leukocyte associated pathways previously seen (Fig. 4C).

An MCL-clustered PPI network of the overlapping proteins between the prioritised sets revealed five distinct clusters (Fig. 4D). These clusters prominently featured T-cell receptor–associated proteins, including those linked to primary immunodeficiency and ZAP-70 translocation, as well as proteins involved in mitosis. The PPI network also highlighted B-cell receptor signalling pathways which was absent from either dataset when analysed independently, alongside IL-1 signalling, which was shared across both datasets.

## Discussion

The clinical and biological heterogeneity of BPD presents challenges for identifying the molecular processes that drive progression of the disease. To address this, we applied a validated in silico approach to systematically integrate and prioritise existing genome-scale BPD datasets from both human and rodent studies. Further to this, we compared our human BPD MAIC results to our previously generated ARDS MAIC dataset to identify conserved mechanisms of lung injury.

Components of the immunological synapse are represented on both sides of the interaction landscape. Consistent across all three gene sets is the enrichment of genes involved in leukocyte-mediated antigen presentation as well as lymphocyte development and activation. Notably, CD3E (CD3ε) emerges as a core node within each protein–protein interaction (PPI) hub, despite its relatively low ranking in the MAIC analyses (human rank = 787; rodent rank = 1631). CD3ε is an essential component of the CD3 complex, which participates in multiple intracellular signal transduction pathways, while CD3ε itself may play a critical role in T-cell development and proliferation [62, 63].

CD3ε can also play a key role in T-cell activation. Evidence of T-cell activation is further supported by the presence of ICOS and CD28 across the identified PPI hubs. Together, these findings suggest a role for modulation of T-cell activation, potentially influencing developmental processes, in the pathogenesis of BPD and related disorders.

A recent study of potential transcriptomic endotypes in BPD focused on the Pietrzyk dataset included in our analysis [12, 35] and identified 4 potential endotypes underlying BPD progression. T helper 17 (Th17) differentiation emerged as the most significant pathway distinguishing the BPD endotypes. CD4 + T-cells were shown to be reduced in infants who develop BPD [64]. It has also been observed that frequencies of populations of CD4 + T-cells are increased preceding the development of BPD [65]. In adults with a history of BPD, a lower CD4/CD8 ratio has been measured. These independent observations and our integrated results suggest that progression of Bronchopulmonary Dysplasia is accompanied by marked effects on T-cell development and activation.

Comparison of the BPD and ARDS MAIC analyses showed that 12% of the prioritised BPD gene set overlapped with the ARDS gene set. This modest overlap suggests there is divergence in underlying disease mechanisms. However, the overlapping genes represent a conserved signature of severe respiratory failure. Our comparative analysis of the ARDS and BPD gene sets further underscored the involvement of lymphoid cells in ventilator-associated injury, with enrichment of pathways related to leukocyte activation, antigen presentation, and innate–adaptive immune crosstalk. Notably, pathways associated with lymphocyte differentiation did not show enrichment in the ARDS MAIC data set suggesting the developmental context of BPD induces specific effects on adaptive immune maturation.

The primary goal of carrying out parallel MAIC analyses between human and animal model BPD studies was to identify core conserved disease mechanisms and differentiate these from species-specific responses to injury in animal models. The rodent MAIC analysis showed a more pronounced focus on pathways associated with extracellular matrix remodelling, and wound healing (Fig. 4A). This divergence is multifactorial but may be influenced by the sample source bias inherent in the existing literature. Over half of the human studies used circulating blood samples (52.4%), which is ideal for capturing systemic immune and biomarker signals. Conversely, 95% of the included rodent studies used whole lung tissue, directly capturing the local pathology and the extensive tissue remodelling inherent to the structural component of BPD. Two of our included human studies use lung tissue as their sample source, restricting a tissue-specific cross-species comparison using MAIC. Interpretation of our findings should consider that many commonly used rodent models of BPD, particularly hyperoxia-based models, predominantly mimic structural features such as impaired alveolarisation and vascular development, while incompletely modelling the full functional and biological complexity of human BPD [4].

Despite the difference in their systemic and local pathologies, there is notable convergence in the human and rodent signals. This is demonstrated by the shared presence of Interleukin-1 Receptor Type 2 (IL1R2) in the top ranked genes of the human (rank = 3) and rodent (rank = 3) datasets. Additional evidence for IL-1 signalling involvement is provided by the enrichment of IL-1–associated clusters within PPI hubs across our comparative analyses. IL1R2 is upregulated in acute hypoxia, [66] and functions as a decoy receptor, acting as a crucial endogenous brake on the potent pro-inflammatory signalling cascade initiated by Interleukin-1 (IL-1). The preterm lung is uniquely vulnerable to hypoxia, which can trigger a dangerous, self-amplifying cycle of injury and inflammation [67]. IL-1 antagonism has been shown to confer protection in rodent models of BPD, [68, 69] suggesting that IL1R2 exerts a protective role as an anti-inflammatory ‘brake’ protein in this context.

This study is based on the published literature and is intended to capture the current state of genome-scale BPD research. It is subject to several limitations. Firstly, blood is among the least invasive and most practical biological samples to collect in the Neonatal Intensive Care setting. The studies included in our human MAIC analysis were largely transcriptomic and predominantly analysed blood-derived samples collected at birth or in the early postnatal period, therefore prioritised gene lists may represent systemic immune and inflammatory processes, and may underrepresent lung-specific disease mechanisms that occur during foetal development. As detailed above, this difference in sample source for human and rodent data represents a primary limitation on direct comparability and interpretation of tissue-specific pathology. We considered performing a subgroup MAIC analysis restricted to human lung tissue to allow for direct comparison with the rodent data; however, only two human studies were available. Application of the MAIC algorithm to a set of two studies would not yield statistically meaningful results, and alternative methods would need to be used to address this limitation. Second, owing to experimental design constraints, the rodent BPD models included in this analysis may be disproportionately weighted toward the effects of hyperoxia on the developing lung, rather than capturing the full complexity of the disease. Third, inherent limitations of cross-species genetic analyses arise from imperfect gene mapping and the absence of direct gene counterparts between species. While the inclusion of the ARDS dataset provided a filter for identifying core lung injury mechanisms, we acknowledge the significant aetiological and clinical differences between adult respiratory distress and neonatal BPD. Finally, MAIC does not account for direction of effect, thus we are limited on how much we can infer about specific mechanisms [70].

## Conclusions

In summary, MAIC offers a validated, integrative strategy capable of navigating the substantial clinical and molecular heterogeneity that characterises BPD. Through this framework, we defined sets of consensus genes across diverse genome-scale datasets. Notably, the aggregated signal is concentrated within lymphoid lineage signatures, and underscores key processes involved in antigen presentation, T-cell activation and development.

## Supplementary Information

Supplementary Material 1.

## Data Availability

All data generated or analysed during this study are included in this published article and its supplementary information files. The MAIC output is included as supplementary material. All code is available at https://github.com/baillielab/bpd_maic.

## Abbreviations

ARDS: Acute Respiratory Distress Syndrome
BPD: Bronchopulmonary Dysplasia
ExomeSeq: Exome Sequencing
GA: Gestational Age
GO: Gene Ontology
GSEA: Gene Set Enrichment Analysis
GWAS: Genome-Wide Association Study
IUGR: Intrauterine Growth restriction
LPS: Lipopolysaccharide
MAIC: Meta-Analysis of Information Content
MassSpec: Mass Spectrometry
MCL: Markov Clustering
Oral: Oral mucosa
ORA: Over-Representation Analysis
PPI: Protein-Protein Interaction
QTL: Quantitative Trait Loci
Resp. Supp: Respiratory Support
RNAseq: Ribonucleic Acid Sequencing
scRNASeq: Single-cell RNAseq
UCB: Umbilical Cord Blood
