# Supplementary material for: Systems-level insights into bronchopulmonary dysplasia from meta-analysis of genome-scale studies
Source: Respir Res. 2026 Jul 15;27:400. doi: 10.1186/s12931-026-03787-z (PMC13670666; doi:10.1186/s12931-026-03787-z)
Supplement: Supplementary file 1 [file 12931_2026_3787_MOESM1_ESM.zip › 26_happs_khanna_supplementary_revised.docx]

**Systems-Level Insights into Bronchopulmonary Dysplasia from Meta-Analysis of Genome-Scale Studies**

Chris Happs^1,2^*, Prerna Khanna^1,2^*, Charlotte Scoynes^2^, Nelly Muriungi^1^, Akira J. T. Alexander^1^, Josh A. Rogers^1^, Elizabeth Lake^1,2^, Maximilian Pickup^1,2^, Nicholas Parkinson^1,3^, Jonathan Millar^2^, J. Kenneth Baillie^1,2^, Sara Clohisey Hendry^1,2^

* These authors contributed equally

1. Roslin Institute, University of Edinburgh, Edinburgh, U.K.
2. Baillie-Gifford Pandemic Science Hub, Institute for Regeneration and Repair, University of Edinburgh, Edinburgh, U.K.
3. Royal (Dick) School of Veterinary Studies, University of Edinburgh, Edinburgh, U.K.

**Supplementary Materials**

Supplementary Tables

**Supplementary Table 1**

| **Inclusion** | **Exclusion** |
| --- | --- |
| Bronchopulmonary Dysplasia,  Hyaline Membrane Disease,  Respiratory Distress Disorder | Meta-analyses, *in-silico* analyses, or re-analysis of previously published data |
| Accepted methodologies | Candidate *in-vivo* or *in-vitro* transcriptomic or proteomic studies (defined as those investigating < 50 genes) |
|  | Candidate gene association studies |
| **Human** | |
| *In vivo, in vitro*, primary human cells and tissues | Term neonates, Children, Adults |
| Any/all treatment administered to patients before sample collection |  |
| **Animal** | |
| Mammals (non-human) |  |
| *In vivo* studies |  |
| Hyperoxia, induced IUGR, LPS treatment |  |

Supplementary Table 1: Inclusion and exclusion criteria for MAIC analysis

**Supplementary Table 2**

| Accepted Methodologies | MAIC category |
| --- | --- |
| CRISPR screen | CRISPR |
| RNAi screen | RNAi |
| Protein-protein interaction study | Protein-protein |
| Genome-wide association study | GWAS |
| Transcriptomic study | RNAseq, scRNAseq, Microarray, |
| Proteomic study | Mass Spec |
| Selected gene-set screens | Geneset |
| Exome sequencing | Exomeseq |
| Epigenetic study (e.g. DNA methylation) | DNAMethylation |

# Supplementary Table 2: Methodologies accepted for inclusion and associated labels/categories for MAIC analysis.

**Supplementary Table 3**

| **Human MAIC analysis** | | **Animal MAIC analysis** | |
| --- | --- | --- | --- |
| **Technique** | **Number of studies** | **Technique** | **Number of studies** |
| Microarray | 6 | Microarray | 11 |
| ExomeSeq | 4 | RNAseq | 18 |
| RNAseq | 5 | GWAS | 1 |
| GWAS | 4 | scRNAseq | 4 |
| Mass Spec | 2 | DNA methylation | 1 |

Supplementary Table 3: Methodologies used in included studies.

**Supplementary Table 6**

| **Dataset Comparison** | **Significance** |
| --- | --- |
| ARDS human gross versus BPD human gross |  |
| ARDS human priority versus BPD human priority | *** |
| BPD human gross versus BPD rodent gross |  |
| BPD human priority versus BPD rodent priority | *** |

Supplementary Table 6: Significance of overlaps between ranked gene lists. * = p<0.05, ** = p<0.005, *** = p<0.0005, - = p>0.05.

**Supplementary Methods**

***Search Strategy***

The search strategy used to identify relevant literature for the systematic review:

1. "Bronchopulmonary Dysplasia*".ti,ab,kf,kw.

2. "Respiratory Distress Syndrome, Newborn*".ti,ab,kf,kw.

3. "Hyaline Membrane Disease*".ti,ab,kf,kw.

4. 1 or 2 or 3

5. "gene*".mp.

6. "genome*".mp.

7. "transcript*".mp.

8. "protein*".mp.

9. 5 or 6 or 7 or 8

10. 4 and 9

11. (Letter or Conference Abstract or Conference Paper or Conference Review or Editorial or Erratum or Review or Note or Tombstone).pt.

12. 10 not 11

***Overlap Analysis of Ranked Gene Lists***

To assess the degree of similarity between ranked lists, we computed the percent overlap at incremental list lengths. Beginning with the top-ranked gene from each list, we iteratively increased the comparison window by one gene until reaching a maximum of n genes. At each step, the proportion of overlapping genes was calculated as the number of shared genes divided by the number of genes included at that step, expressed as a percentage.

**Supplementary Figure 1:**

**Identification of studies via databases and registers**

Records identified from:

Databases (n = 6831)

- *Medline (n = 2624)*
- *Embase (n = 3907)*

Registers (n = 0)

Records removed *before screening*:

Duplicate records removed (n = 2081)

Records marked as ineligible by automation tools (n = 0)

Records removed for other reasons (n = 0)

**Identification**

Records screened

(n = 4450)

Records excluded by humans

(n =4369)

Reports sought for retrieval

(n = 81)

Reports not retrieved

(n = 5)

**Screening**

Reports excluded:

Not methodology of interest (n = 16)

Insufficient data or sample size (n = 7)

Poster or conference abstract (n = 32)

Reports assessed for eligibility

(n = 76)

Studies included in review

(n = 21)

Reports from included studies

(n = 31)

**Included**

*From:*  Page MJ, McKenzie JE, Bossuyt PM, Boutron I, Hoffmann TC, Mulrow CD, et al. The PRISMA 2020 statement: an updated guideline for reporting systematic reviews. BMJ 2021;372:n71. doi: 10.1136/bmj.n71

**Supplementary Figure 2**

**Identification of studies via databases and registers**

Records identified from:

Databases (n = 6831)

- *Medline (n = 2624)*
- *Embase (n = 3907)*

Registers (n = 0 )

Records removed *before screening*:

Duplicate records removed (n = 2081)

Records marked as ineligible by automation tools (n = 0)

Records removed for other reasons (n = 0 )

**Identification**

Records screened

(n = 4450 )

Records excluded

(n =4313)

Reports sought for retrieval

(n =137)

Reports not retrieved

(n = 19)

**Screening**

Reports excluded:

- Not methodology of interest (n = 31)
- Insufficient data or sample size (n = 14)
- Poster or conference abstract (n = 43)
- Duplicate (n = 2)
- Raw data only available (n = 8)

Reports assessed for eligibility

(n = 118)

Studies included in review

(n =20)

Reports of included studies

(n = 35)

**Included**

*From:*  Page MJ, McKenzie JE, Bossuyt PM, Boutron I, Hoffmann TC, Mulrow CD, et al. The PRISMA 2020 statement: an updated guideline for reporting systematic reviews. BMJ 2021;372:n71. doi: 10.1136/bmj.n71

Supplementary Figure 3

**Supplementary Figure 3:** A) Heatmap of top 100 ranked genes based on their evidence for a role in BPD progression in human studies. Genes are ranked (y-axis) by MAIC score. The source of the data is indicated on the x-axis. B) Circos representation of shared information content among data sources in the MAIC analysis. Each experiment or data source is represented by a block on the outer ring of the circle; the size of data source blocks is proportional to the summed information content of the input list—that is, the total contribution that this data source makes to the aggregate, calculated as the sum of the MAIC gene scores contributed by that list. Lines are coloured according to the dominant data source. Data sources within the same category share the same colour. C) Enrichment plot of top pathways in Figure 1 B. D) Gene prioritisation plot using the invariant knee method. The inflection point is indicated with a dotted line. 945 genes in the left upper quadrant of the knee threshold were prioritised for downstream analysis.

Supplementary Figure 4

**Supplementary Figure 4:** Top 20 general cell types for the prioritised human BPD gene set, as indicated by WebCSEA.

Supplementary Figure 5

**Supplementary Figure 5:** A-E) Pathway analysis using String db of prioritised human BPD gene set. F) UpSet plot of overlapping proteins indicating hub proteins, identified by five methods: MNC - Maximum Neighbourhood Component, DMNC - Density of MNC, EPC - Edge Percolated Component, MCC - Maximal Clique Centrality.

Supplementary Figure 6

**Supplementary Figure 6:** A) Percent overlap between the top 50 MAIC-ranked Human ARDs genes versus the top 50 Human BPD genes. B) Percent overlap between the top 50 MAIC-ranked Human BPD genes versus the top 50 Rodent BPD genes.

Supplementary Figure 7

**Supplementary Figure 7:** A) Heatmap of top 100 ranked genes based on their evidence for a role in BPD progression in human studies. Genes are ranked (y-axis) by MAIC score. B) Representation of shared information content among data sources in the MAIC analysis. Each experiment or data source is represented by a block on the outer ring of the circle; the size of data source blocks is proportional to the summed information content of the input list—that is, the total contribution that this data source makes to the aggregate, calculated as the sum of the MAIC gene scores contributed by that list. Lines are coloured according to the dominant data source. Data sources within the same category share the same colour. C) Enrichment plot of top pathways in Figure 3 B. D) Gene prioritisation plot using the invariant knee method. The inflection point is indicated with a dotted line. 1783 genes in the left upper quadrant of the knee threshold were prioritised for downstream analysis.

Supplementary Figure 8

**Supplementary Figure 8:** Top 20 general cell types for the prioritised rodent BPD gene set, as indicated by WebCSEA.

Supplementary Figure 9

**Supplementary Figure 9:** A-E) Pathway analysis using String db of prioritised rodent BPD gene set. F) UpSet plot of overlapping proteins indicating hub proteins, identified by five methods: MNC - Maximum Neighbourhood Component, DMNC - Density of MNC, EPC - Edge Percolated Component, MCC - Maximal Clique Centrality.
