# Supplementary material for: Systems-level insights into bronchopulmonary dysplasia from meta-analysis of genome-scale studies
Source: Respir Res. 2026 Jul 15;27:400. doi: 10.1186/s12931-026-03787-z (PMC13670666; doi:10.1186/s12931-026-03787-z)
Supplement: Supplementary file 1 [file 12931_2026_3787_MOESM1_ESM.zip › Supplementary Methods.docx]

Supplementary Methods

The search strategy used to identify relevant literature for the systematic review:

1. "Bronchopulmonary Dysplasia*".ti,ab,kf,kw.

2. "Respiratory Distress Syndrome, Newborn*".ti,ab,kf,kw.

3. "Hyaline Membrane Disease*".ti,ab,kf,kw.

4. 1 or 2 or 3

5. "gene*".mp.

6. "genome*".mp.

7. "transcript*".mp.

8. "protein*".mp.

9. 5 or 6 or 7 or 8

10. 4 and 9

11. (Letter or Conference Abstract or Conference Paper or Conference Review or Editorial or Erratum or Review or Note or Tombstone).pt.

12. 10 not 11
