# Supplementary material for: Systems-level insights into bronchopulmonary dysplasia from meta-analysis of genome-scale studies
Source: Respir Res. 2026 Jul 15;27:400. doi: 10.1186/s12931-026-03787-z (PMC13670666; doi:10.1186/s12931-026-03787-z)
Supplement: Supplementary file 1 [file 12931_2026_3787_MOESM1_ESM.zip › supp_figure_2.docx]

**Supplementary Figure 2**

**Identification of studies via databases and registers**

Records identified from:

Databases (n = 6831)

- *Medline (n = 2624)*
- *Embase (n = 3907)*

Registers (n = 0 )

Records removed *before screening*:

Duplicate records removed (n = 2081)

Records marked as ineligible by automation tools (n = 0)

Records removed for other reasons (n = 0 )

**Identification**

Records screened

(n = 4450 )

Records excluded

(n =4313)

Reports sought for retrieval

(n =137)

Reports not retrieved

(n = 19)

**Screening**

Reports excluded:

- Not methodology of interest (n = 31)
- Insufficient data or sample size (n = 14)
- Poster or conference abstract (n = 43)
- Duplicate (n = 2)
- Raw data only available (n = 8)

Reports assessed for eligibility

(n = 118)

Studies included in review

(n =20)

Reports of included studies

(n = 35)

**Included**

*From:*  Page MJ, McKenzie JE, Bossuyt PM, Boutron I, Hoffmann TC, Mulrow CD, et al. The PRISMA 2020 statement: an updated guideline for reporting systematic reviews. BMJ 2021;372:n71. doi: 10.1136/bmj.n71
